# Analysis of the Antioxidant Composition of Low Molecular Weight Metabolites from the Agarolytic Bacterium *Alteromonas macleodii* QZ9-9: Possibilities for High-Added Value Utilization of Macroalgae

**DOI:** 10.3390/antiox11101977

**Published:** 2022-10-03

**Authors:** Xinyi Wang, Ziqiao Feng, Chenhui Li, Xiaoni Cai, Hao Long, Xiang Zhang, Aiyou Huang, Yanhua Zeng, Wei Ren, Zhenyu Xie

**Affiliations:** 1 State Key Laboratory of Marine Resource Utilization in the South China Sea, Hainan University, Haikou 570228, China; 2 Hainan Provincial Key Laboratory for Tropical Hydrobiology and Biotechnology, Hainan University, Haikou 570228, China; 3 College of Marine Sciences, Hainan University, Haikou 570228, China; 4 Laboratory of Development and Utilization of Marine Microbial Resource, Hainan University, Haikou 570228, China

**Keywords:** agarolytic bacterium, macroalgae, agarase, antioxidant activity, antioxidant peptides, maculosin

## Abstract

Agar accounts for ~60% of the dry weight of some red macroalgae, and the breakdown of this kind of polysaccharide releases high-value compounds; therefore, the resource utilization of agar is of great significance to improve the added value of these macroalgae. Herein, *Alteromonas macleodii* QZ9-9 isolated from tropical *Gracilaria hainanensis* in Hainan Island was characterized as an agarolytic bacterium, which displayed a high agar-degrading activity. The highest diameters of the degradation zones of the *A. macleodii* QZ9-9 and its extracellular-agarase (12.16 U/mL) were 41.46 mm and 22.89 mm, respectively, and the first-order degradation rate constants of those were 0.02 h^−1^ and 0.77 U^−1^, respectively. Importantly, the fermentation products of *A. macleodii* QZ9-9 exhibited antioxidant activity, and the peak of DPPH scavenging activity of 50 h fermentation products of this strain was up to 50.79% in the reaction for 1 h; the DPPH scavenging activity of low molecule metabolites (≤3 kDa) in particular was up to ~85.85%. A total of 766 metabolites were detected in the low molecule metabolites by metabolomics. The peptide-like metabolites, such as prolyl–histidine, isoleucyl–histidine, isoleucyl–proline and arginyl–proline, and the antioxidant maculosin were found in the top 20 metabolites with relatively high abundance. Additionally, the antioxidant activity of maculosin was further verified in this work. We concluded that the low molecule metabolites of *A. macleodii* QZ9-9 with relatively high antioxidant activity are interesting candidates for preparing desirable non-toxic antioxidants, thereby facilitating the high value-added utilization of macroalgae in the fields of cosmetic, food preservation, and pharmaceutical industries.

## 1. Introduction

Macroalgae are commonly divided into three subclassifies, including green algae (Chlorophyta), brown algae (Phaeophyta), and red algae (Rhodophyta), and found in abundance along the coast in intertidal zones, sublittoral-to-littoral zones, and lighted rock or other hard substrata [1]. Agar, as a main heterogeneous hydrocolloid polysaccharide compound of red algae (such as *Gelidium*, *Gracilaria*, and *Ceramium*), mainly consists of agarose and agaropectin and is commonly employed as a food additive and gelling agent for bacterial culture with a huge production of 10,600 tons per year [2,3]. However, the potential highly valuable bioactive of the agar of these macroalgae species has not given full play to its function related to the properties of high viscosity, low water solubility, and difficulty in being absorbed.

Reportedly, numerous agarolytic bacteria could metabolize agar by the agarase secretion [3], which are ubiquitous in marine environments, such as *Vibrio*, *Pseudomonas*, *Pseudoalteromonas*, *Streptomyces*, *Alteromonas*, *Microbulbifer*, etc. [4,5]. More importantly, the agarases produced by agarolytic bacteria can convert agar into high-value chemicals such as ethanol, butanol, and various oligosaccharides [6]. Of the high-value chemicals, the oligosaccharides are reported as prebiotics with the advantageous properties of anti-inflammatory, antibacterial, whitening effect, immune modulation, and antitumor and antioxidant activities [7,8,9,10,11,12,13,14]. Therefore, the high-value utilization of macroalgae by degrading its agar has become an existing research hotspot. 

Currently, the main methods of agar degradation are chemical and bio-degradation, whereas the research in the preparation of high-value compounds from macroalgae agar has been devoted toward bio-degradation because of its high specificity, mild conditions, and no secondary pollution [15]. However, it is worth noting that most reports about agarolytic bacteria solely focus on their agarase and the enzymatic hydrolysis products of this species, while few works have comprehensively investigated the metabolites of these agarolytic bacteria. Agarolytic bacteria have many points of concern, for instance, several agarolytic bacteria of *Vibrio*-like species have been regarded as nitrogen fixers for anaerobic growth using agar as the sole source of carbon and energy [14,16], and the agarolytic extreme thermophile *Caldicellulosiruptor saccharolyticus* displayed the capacity of producing hydrogen by degradation of natural polysaccharides agar [17].

*Alteromonas macleodii* QZ9-9 (CGMCC No. 24568), identified as an agarolytic bacterium, was isolated from tropical *G. hainanensis*, and the low molecule fermentation products (≤30 kDa) of this strain exhibited relatively high antioxidant activity. In this work, the agar-degrading capacity of *A. macleodii* QZ9-9 and analysis of the antioxidant components in the metabolites of this agarolytic bacterium were investigated, thereby facilitating the high value-added utilization of macroalgae in the future.

## 2. Materials and Methods

### 2.1. Screening and Identification of Agarolytic Bacteria

The fresh *G. hainanensis* samples were collected from the coast of Hainan Island, China, ground, and centrifuged at 8000 rpm for 10 min in turn to obtain the bacterial mixture. Subsequently, the bacterial mixture was used to enrich the agarolytic bacteria in an agar-enriched medium (agar as the sole source of carbon and energy; w/v: containing 0.12% agar, 3% NaCl, 0.5% (NH_4_)_2_SO_4_, 0.2% K_2_HPO_4_, 0.1% MgSO_4_·7H_2_O and 0.01% FeSO_4_·7H_2_O, pH 7.5). The incubation was carried out at 30 °C in a shaking incubator at 180 rpm for 48 h. After several subcultures, the enrichment culture was serially diluted and spread onto mineral salts agar plates (*w*/*v*: containing 0.2% agar, 3% NaCl, 0.5% (NH_4_)_2_SO_4_, 0.2% K_2_HPO_4_, 0.1% MgSO_4_·7H_2_O and 0.01% FeSO_4_·7H_2_O, pH 7.5) and incubated at 30 °C for 48 h. The individual colonies with obvious degradation zones were subcultured on fresh plates to isolate pure agarolytic bacteria.

The strain QZ9-9 with efficient agar-degrading capacity was isolated and characterized as an agarolytic bacterium, and its genomic DNA was extracted using the bacterial genomic DNA extraction kit (TIANGEN Biotech Co., Ltd., Beijing, China). The 16S rRNA gene was amplified from the genomic DNA by PCR using the primers 27F (5′-AGAGTTTGATCCTGGCTCAG-3′) and 1492R (5′-TAGGCTACCTTGTTACGACTT-3′), and the amplified fragment of 1382 bp was obtained. PCR products were sequenced, and 16S rRNA homological gene sequences were searched by the BLAST program in the GenBank database (https://blast.ncbi.nlm.nih.gov/Blast.cgi?CMD=Web&PAGETYPE=BLASTHome; accessed on 1 January 2022). The phylogenetic tree was built with the neighbor-joining method using Mega 11.0 software to identify the strain. Additionally, the genome of *A. macleodii* QZ9-9 was deposited in the NCBI database under the accession number CP098772.1.

Additionally, the ultrastructure of *A. macleodii* QZ9-9 was observed by scanning electron microscopy (SEM). Ten microliters of culture broth was spread-plated on sterile glass coverslips, then dried by natural air. The coverslips were immersed in a liquid containing 2.5% (*v*/*v*) glutaraldehyde for fixation. All fixed coverslips were gradually dehydrated by ethanol (ranging from 50 to 100% with a gradient of 10%) for 10 min, then dried by natural air. In turn, the dehydrated coverslips were sputter-coated with gold and viewed by SEM (Verios G4 UC, Thermo Scientific, Waltham, MA, USA).

### 2.2. Detection of Agar-Degrading Activity

The agar-degrading activity of agarolytic bacterium *A. macleodii* QZ9-9 was assessed by the disc diffusion assay. Briefly, 1.5 μL aliquots of a broth culture of QZ9-9 bacterial droplets were plated on the surface of mineral salts agar plates, which were subsequently incubated in a constant temperature incubator (30 °C), and the diameter of the degradation zone was observed every 12 h. Additionally, the agar-degrading activity of crude agarase was also assessed by the disc diffusion assay. Briefly, the strain QZ9-9 was cultivated in an agar-enriched medium at 30 °C under shaking of 180 rpm for 32 h. The fermentation without bacterial cells was used to prepare the crude agarase. The crude enzyme was serially diluted to different enzyme activities and added into Oxford cups (7.8 mm outer diameter × 6 mm inner diameter × 10 mm height) that were previously placed on the mineral salt agar plates. After incubation for 15 h, the diameters of the degradation zones were measured. All the degradation circles were colored with Lugol’s iodine solution (10% (*w*/*v*) KI, 5% (*w*/*v*) I of 100 mL ddH_2_O).

### 2.3. Enzyme Activity Assay

The standard assay of lyase activity was commonly determined by measuring the amount of released reducing sugar equivalent using the 3,5-dinitrosalicylic acid (DNS) method [9,13,14,18,19,20,21,22,23,24]. In this work, the agarase activity was evaluated by the DNS method with some slight modifications. Briefly, the reaction mixture of 1 mL PBS buffer (pH 7.5) containing 0.2% (*w*/*v*) agar and 1 mL crude enzyme solution was incubated at 40 °C for 30 min. In turn, 3 mL DNS was added to the experimental and control mixtures to terminate the reactions, and 1 mL enzyme was added to the control group. The mixture was boiled for 5 min at 100 °C. After cooling to room temperature, the absorbance of the mixture was measured at 540 nm. One unit of agarase activity was defined as the amount of enzyme that released 1 μg of reducing sugar (measured as glucose) from agar in 1 min under the assay condition.

### 2.4. The Curves of Growth and Agarase Production

Strain QZ9-9 was, respectively, inoculated with 2% inoculum for 72 h in a non-agar carbon source medium (*w*/*v*: containing 0.5% tryptone, 0.1% yeast extract, 100 mL seawater, pH 7.5) and a mineral salt medium (w/v: containing 0.2% agar, 3% NaCl, 0.5% (NH_4_)_2_SO_4_, 0.2% K_2_HPO_4_, 0.1% MgSO_4_·7H_2_O, and 0.01% FeSO_4_·7H_2_O, pH 7.5), with agar as the only carbon source. The curves of growth and agarase production of the strain were completely investigated at 600 nm.

### 2.5. Antioxidant Activity Assay

The strain QZ9-9 was aerobically cultured in a non-agar carbon source medium for 20 h at 30 °C with shaking of 180 rpm. Subsequently, 1 mL culture broth was added to 50 mL of the fresh medium to subculture at 30 °C for 6 h. In turn, 10 mL of the above solution was added to 500 mL agar-enriched medium and cultured at 30 °C with shaking of 180 rpm. The samples from different periods of culture broth without bacterial cells were intercepted into the metabolites with different molecular weights by Amicon Ultra Tube (Millipore Sigma, Burlington, MA, USA). The antioxidant activities of ultrafiltrates of different molecular weights were measured at 517 nm with an interval of 1 min using the microplate tester.

The method of antioxidant determination was as follows:

Antioxidant activity was measured by α,α-diphenyl-β-picrylhydrazyl (DPPH) scavenging activity. The DPPH quenching ability was measured by the method proposed by Jiao et al. [25] with slight modifications. Briefly, 50 μL fresh DPPH solution (0.1 mM anhydrous ethanol) was mixed with a 150 μL sample. The mixture reacted in darkness at 25 °C for 1 h, and the absorbance of the reaction solution was measured at 517 nm. Anhydrous ethanol was used as a negative control. The equation of scavenging radical was calculated as follows:

Scavenging Rate (%) = (*A*_0_ − *A*_1_)/*A*_0_ × 100

where *A*_0_ is the absorbance of the control, and *A*_1_ is the absorbance of the sample.

### 2.6. Sample Preparation for Metabolomics of Antioxidant Fermentation

The strain QZ9-9 exhibiting antioxidant properties was aerobically cultured for 50 h in the agar-enriched medium at 30 °C at 180 rpm. After centrifugation, the fermentation products were separated into supernatants with different molecular weight segments by Amicon Ultra Tube (Millipore Sigma, USA). The DPPH scavenging activity of the sample with molecular weight ≤3 kDa (10 mg/mL) was up to 40.88%, so the low molecule metabolites (≤3 kDa) were used in subsequent experiments. The samples with six replicates were then transferred to a new glass vial for metabolic analysis in random order. The quality control samples were prepared by mixing aliquots (20 μL) of each sample and periodically analyzed to monitor the stability of the instrument.

### 2.7. Metabolomics Analysis by UHPLC–QE/MS

The prepared samples were collected for the non-targeted metabolomics analysis using ultra-high-performance liquid chromatography coupled with Q Exactive Orbitrap mass spectrometry (UHPLC-QE/MS; Vanquish Horizon system; Thermo Scientific, Bremen, Germany). ACQUITY UPLC HSS T3 column (2.1 mm × 100 mm × 1.8 m, Waters, Milford, MA, USA) was used to separate metabolites. The chromatography parameters were as follows: flow rate—0.4–0.6 mL/min; sample injection volume—2 μL; gradient mobile phase—water containing 5% acetonitrile and 0.1% formic acid as solvent A, 47.5% acetonitrile, and 47.5% isopropanol containing 0.1% formic acid as solvent B; column temperature—40 °C. The gradient mobile phase program applied was as follows: *t* = 0 min, 100% A, 0.4 mL/min; *t* = 3.5 min, 75.5% A, 0.4 mL/min; *t* = 5 min, 35% A, 0.4 mL/min; *t* = 5.5 min, 0% A, 0.4 mL/min; *t* = 7.4 min, 0% A, 0.6 mL/min; *t* = 7.6 min, 48.5% A, 0.6 mL/min; *t* = 7.8 min, 100% A, 0.5 mL/min; *t* = 9 min, 100% A, 0.4 mL/min; *t* = 10 min, 100% A, 0.4 mL/min. The mass spectrometry parameters were performed in ESI (+): scan type—70–1050 (*m*/*z* 70–1000); sheath gas flow rate—50 arb; auxiliary gas flow rate—13 arb; heater temperature—425 °C; capillary temperature—325 °C; spray voltage—3.5 kV (+); S-Lens RF level—55; resolution—60,000 Full MS and 7500 MS^2^; MS/MS collision energies—20, 40 and 60 eV.

### 2.8. Maculosin Specific Antioxidant Activity

According to the results of the composition analysis of the molecular weight ≤3 kDa, the maculosin was selected to vitrify the antioxidant activity by the method of the DPPH free radical scavenging experiment (the details are presented in the Section 2.5), which was purchased from Shanghai Apeptide Co., Ltd., Shanghai, China. The vitrified concentration of maculosin ranged from 0 μg/mL to 750 μg/mL, and Vitamin C was set as a positive control.

### 2.9. Statistical Analysis

Metabolite data from UHPLC–QE/MS were imported into Progenesis QI software (Waters Corporation, Milford, MA, USA) for nonlinear retention time correction, peak filtration, extraction, and matching. The metabolite molecules and metabolic pathways were further identified by the following databases: HMDB (http://www.hmdb.ca/ (accessed on 9 September 2022)), METLIN (http://metlin.scripps.edu/ (accessed on 9 September 2022)), iPath 3.0 (http://pathways.embl.de (accessed on 9 September 2022)), and KEGG pathway (http://www.genome.jp/kegg/ (accessed on 9 September 2022)). The statistical analyses of other experimental results were performed by SPSS version 17.0 for Windows (SPSS Inc., Chicago, IL, USA), with *p* < 0.05 considered statistically significant differences through one-way analysis of variance (ANOVA) with Dunnett’s post-test.

## 3. Results

### 3.1. Characterization and Identification of Agarolytic Bacteria

**Figure 1:**
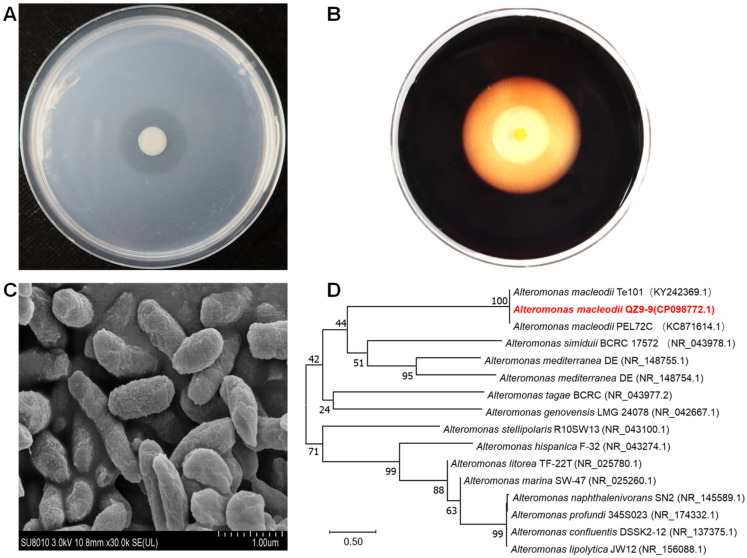
The morphology of agarolytic bacterial *A. macleodii* QZ9-9. (**A**) The colony of strain QZ9-9 on mineral salts agar plate after static incubation for 48 h, and a clear transparent hydrolysis zone surrounds this colony. (**B**) Hydrolysis zone colored with Lugol’s iodine solution after static incubation for 96 h on mineral salts agar plate. (**C**) Close-up of the electron microscopy of strain QZ9-9. (**D**) Phylogenetic tree of *A. macleodii* QZ9-9 (marked by red color) prepared using Mega 11.0 software. Note: the code in each parenthesis represents the sequence accession number in GenBank, and the number at each branch point is the percentage supported by bootstrap.

The strain QZ9-9 from *G. hainanensis* was successfully screened and isolated by the agar-enriched medium with agar as the sole source of carbon and energy for the strain growth and showed a strong agar-degrading capacity according to the results of the disc diffusion assay (Figure 1A,B). The individual colony on the agar plate was circular, entirely convex, smooth, yellowish-white, and with neat edges. The diameter of the hydrolytic zone was approximately 4 mm after incubation for two days at 30 °C (Figure 1A). Strain QZ9-9 was identified as an aerobic Gram-negative bacterium (data not shown), and the ultrastructure of strain QZ9-9 showed that this strain was short rod-shaped and approximately 1.26 μm in length with no flagella (Figure 1C). Meanwhile, according to the whole genome (CP098772.1) and 16S rRNA, strain QZ9-9 was classified into the branch of *Alteromonas macleodii* and named “*Alteromonas macleodii* QZ9-9” (Figure 1D).

### 3.2. The Curves of Growth and Agarase Production of A. macleodii QZ9-9

**Figure 2:**
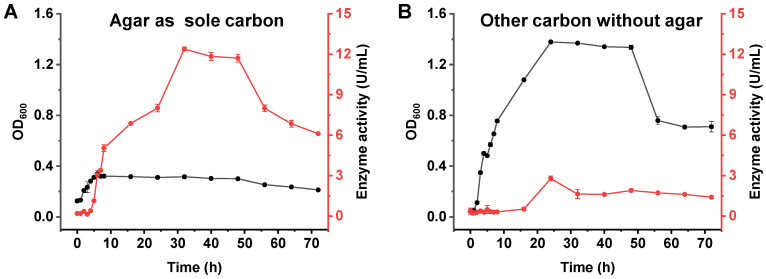
The curves of growth and agarase production of *A. macleodii* QZ9-9 in agar-enriched medium (*w*/*v*: containing 0.2% agar, 3% NaCl, 0.5% (NH_4_)_2_SO_4_, 0.2% K_2_HPO_4_, 0.1% MgSO_4_·7H_2_O and 0.01% FeSO_4_·7H_2_O, pH 7.5) with agar as the sole carbon source (**A**) and agar-deficient medium (*w*/*v*: containing 0.5% tryptone, 0.1% yeast extract, 100 mL seawater, pH 7.5) that other carbon instead of agar as the carbon source (**B**).

The curves of growth and agarase production of *A. macleodii* QZ9-9 were investigated in an agar-enriched medium with agar as the sole carbon source and agar-deficient medium (non-agar carbon source medium), where yeast extract instead of agar was used as the carbon source. As shown in Figure 2, *A. macleodii* QZ9-9 grew better in a non-agar carbon source medium than that in an agar-enriched medium, indicating that the non-agar carbon source medium contained more nutrients than the agar-enriched medium that no other ingredients except agar as carbon source and ions for the growth of strain QZ9-9. It is worth noting that the enzyme-producing capacity of strain QZ9-9 in an agar-enriched medium was better than that in a non-agar carbon source medium (Figure 2), suggesting that the agar must make a great contribution to the agarase production of agarolytic bacteria. Additionally, when strain QZ9-9 was cultured in an agar-enriched medium, the peak enzyme production (about 12.39 U/mL) occurred at 32 h (Figure 2A). However, no matter which medium was used for culturing agarolytic bacterial *A. macleodii* QZ9-9, the growth and enzyme production of this strain showed a short delay period, indicating that *A. macleodii* QZ9-9 had strong environmental adaptability (Figure 2; the red parts).

### 3.3. Analysis of the Agar-Degrading Activity of A. macleodii QZ9-9

The agar-degrading capacities of *A. macleodii* QZ9-9 and agarase secreted by this strain were completely investigated with the disc diffusion assay.

**Figure 3:**
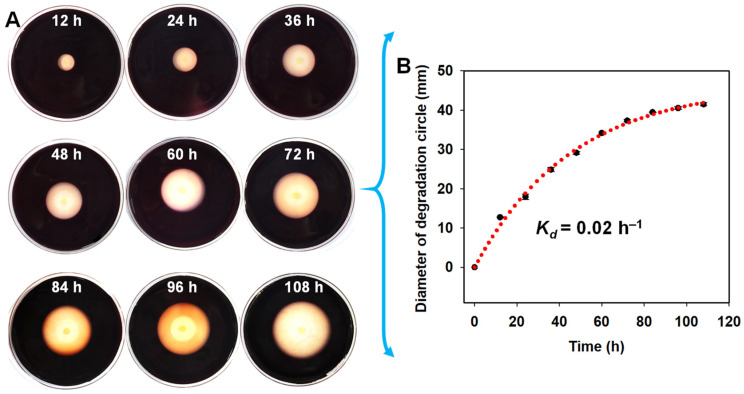
The agar-degrading capacity of *A. macleodii* QZ9-9. (**A**) Characterization of agar-degrading activity of *A. macleodii* QZ9-9 on the mineral salts agar plates (stained with Lugol’s iodine solution) at different degradation periods. (**B**) Kinetics of agar degradation by *A. macleodii* QZ9-9. Note: The data were fitted according to the first-order rate equation *Y_t_* = *Y*_0_ + *a*(1 − e^−*k*x^), where *Y_t_* and *Y*_0_ are the degradation fraction at a given culture time *t* and 0 min, respectively; *a* is the fitting coefficients; and *k* is the observed rate constant.

As shown in Figure 3, the diameter of the degradation zone generated by the agarolytic bacterium kept increasing with the increase in culture time (Figure 3A), and the first-order degradation rate constant was about 0.02 h^−1^ (Figure 3B). The maximum degradation diameter of 41.46 mm of the strain QZ9-9 occurred at 108 h. Additionally, the degradation growth rate of strain QZ9-9 was maintained at a relatively high level in the period from 0 h to 60 h, whereas that tended to be gentle after 60 h (Figure 3B). 

**Figure 4:**
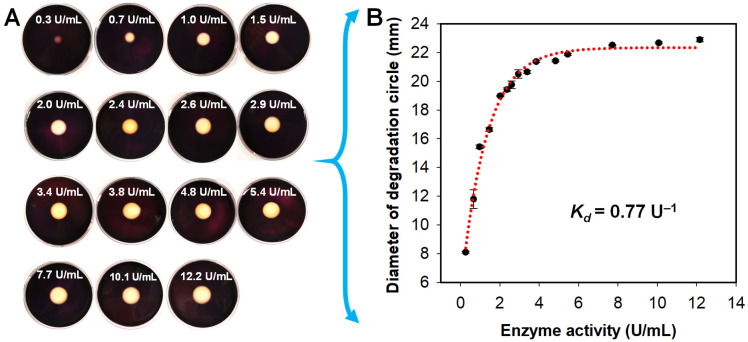
The agar-degrading capacities of agarase secreted by *A. macleodii* QZ9-9. (**A**) Characterization of agar-degrading activity of crude agarase with different enzyme activities on the mineral salts agar plates (stained with Lugol’s iodine solution). (**B**) Kinetics of agar degradation by agarase. Note: The data were fitted according to the first-order rate equation *Y_EA_* = *Y*_0_ + *a*(1 − e^−*k*x^), where *Y_EA_* and *Y*_0_ are the degradation fraction at a given enzyme activity *EA* and 0 U/mL, respectively; *a* is the fitting coefficients; and *k* is the observed rate constant.

As shown in Figure 4, the diameter of the degradation zone generated by agarase increased with the increase in enzyme activity. The results showed that the agar-degrading ability of agarase was considerable (Figure 4A), and the first-order degradation rate constant was about 0.77 U^−1^ (Figure 4B). At the same time, the degradation growth rate of agarase was maintained at a high level at the enzyme activity ranging from 0 to 3.84 U/mL, while that tended to be gentle when the agarase was higher than 3.84 U/mL. Meanwhile, the maximum degradation diameter of 22.89 mm of agarase activity was about 12.2 U/mL.

### 3.4. Analysis of the Antioxidant Activity of Fermentation Products of A. macleodii QZ9-9

**Figure 5:**
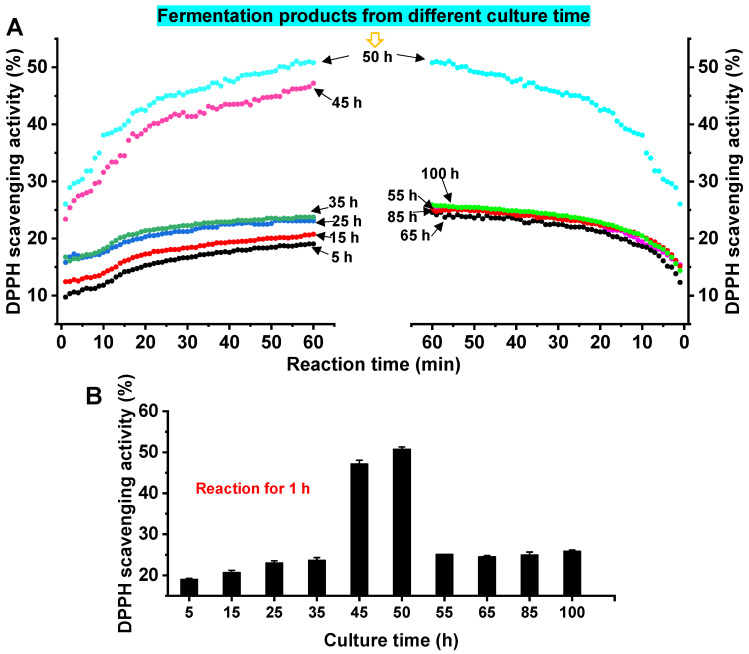
Kinetics of DPPH scavenging activity of fermentation products from two culture periods during the reaction time of 1 h, (**A**) the period of 0 h to 50 h, and (**B**) the period of 50 h to 100 h. (**C**) DPPH scavenging activity of fermentation products during the culture of strain QZ9-9 after 1 h reaction. Note: The reaction time refers to the time consumed by the combination reaction between fermentation products and DPPH solution.

After a lot of preliminary explorations of the function of *A. macleodii* QZ9-9, we found that the agarolytic bacterial fermentation products without cells displayed a strong antioxidant property. Therefore, the antioxidant properties of the *A. macleodii* QZ9-9 fermentation products were characterized by the method of the DPPH free radical scavenging experiment. The kinetics of antioxidant properties of fermentation products (10 mg/mL) from different culture periods are regularly changed. As shown in Figure 5, the DPPH scavenging activity of fermentation products from each fermentation period increased with the increase in reaction time, and the scavenging rate increased rapidly in the period of 0 min to 23 min, indicating that the fermentation products can quickly react with DPPH. Additionally, the DPPH scavenging activity of fermentation products increased with the time increase during the fermentation period of 5 h to 50 h, and the scavenging activity reached the highest when the fermentation time was 50 h (Figure 5A). However, when the fermentation time exceeded 50 h, the DPPH clearance rate decreased sharply during the fermentation period of 50 h to 65 h, then tended to be flat (Figure 5B). We reason that the occurrence of this phenomenon depended on the characteristics of the growth and enzyme production of agarolytic bacterial strain *A. macleodii* QZ9-9. *A. macleodii* QZ9-9 had been growing and continuously producing agarase leading to more productions of antioxidant substances including metabolites and degradants during the culture of 50 h, whereas the growth and the enzyme production capacities of this strain gradually decreased leading to the decline and decomposition of the production of bioactive substances (particularly antioxidant products) after culture of 50 h. Additionally, as shown in Figure 5C, the fermentation broths of strain QZ9-9 from the culture period of 40 h to 50 h exhibited high DPPH scavenging activity, and the scavenging rate of DPPH reached the maximum of 50.79% at 50 h. 

**Figure 6:**
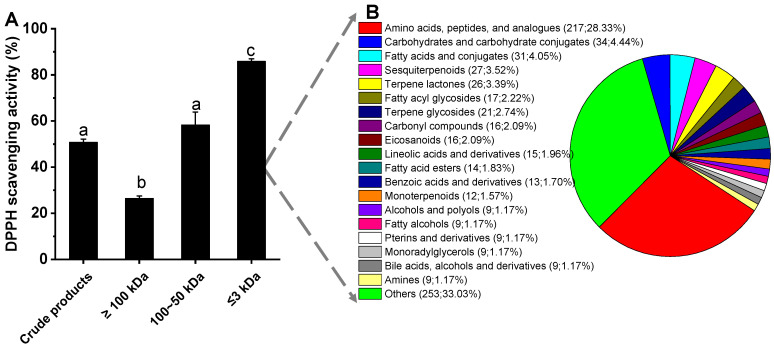
(**A**) The DPPH scavenging activity of fermentation products with different molecular weights. (**B**) Comparison of the relative abundance of metabolites in the low molecular weight (≤3 kDa) products. The different superscript letters indicate a significant difference at *p* < 0.05.

According to the above results, the fermentation products (without bacterial cells) of strain QZ9-9 from the culture broth of 50 h were used for further analysis of antioxidant composition. As shown in Figure 6A, the fermentation broth with a molecular weight of less than 3 kDa showed relatively high antioxidant activity, and its antioxidant activity increased with the increase in its concentration. Therefore, we reason that there are certain amounts and types of antioxidants in the low molecule metabolites (≤3 kDa) of *A. macleodii* QZ9-9 after incubation for 50 h.

### 3.5. Overview of the Composition of the Low-Molecule Metabolites

A comprehensive understanding of the reasons why low molecular weight (≤3 kDa) fermentation broth of *A. macleodii* QZ9-9 exhibited relatively high antioxidant activity will lay a foundation for the subsequent separation and utilization of bioactive substances from this strain. In this section, through the method of metabolomics, we completely investigated the global composition of the low molecular weight fermentation products of *A. macleodii* QZ9-9, which exhibited relatively high DPPH scavenging activity. A total of 766 metabolites in positive ion mode were detected in the low molecular weight (≤3 kDa) fermentation broth (Figure 6B; the detail of metabolomics is shown in Supplementary Materials), and there were 219 metabolites annotated in KEGG and 555 metabolites annotated in the database of HMDB. All the low molecular weight (≤3 kDa) were divided into chemical categories as 217 amino acids/peptides/analogs, 34 carbohydrates/carbohydrate conjugates, 31 fatty acids/conjugates, 27 sesquiterpenoids, 26 terpene lactones, 17 fatty acyl glycosides, 21 terpene glycosides, 16 carbonyl compounds, 16 eicosanoids, 15 lineolic acids/derivatives, 14 fatty acid esters, 13 benzoic acids/derivatives, 12 monoterpenoids, 9 alcohols/polyols, 9 fatty alcohols, 9 pterins/derivatives, 9 monoradylglycerols, 9 bile acids/alcohols/derivatives, and 9 amines. The metabolites of the low molecular weight fermentation products of *A. macleodii* QZ9-9 with the relative abundances of the top 20, such as 54.96% meso–pristane, 2.68% prolyl–histidine, 1.58% C16 sphinganine hexadecasphinganine, 1.20% glycerol, 1.12% 6-hydroxyoctadecanoic acid, and 0.95% maculosin, are listed in Table 1.

### 3.6. Maculosin Specific Antioxidant Activity

**Figure 7:**
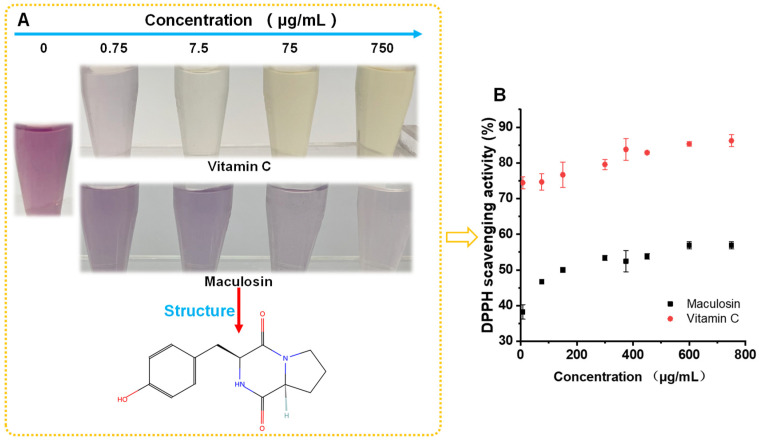
Confirmation of DPPH scavenging activity of maculosin by visual observation (**A**) and quantification (**B**). Vitamin C is a positive control.

Due to the relatively high content of maculosin, which was reported to have antioxidant properties in the low molecular weight (≤3 kDa) of *A. macleodii* QZ9-9, the DPPH scavenging activity of maculosin was verified in this work. If the substance possesses antioxidant properties, it can fade the purple color of the DPPH solution. As shown in Figure 7, the DPPH scavenging activity of maculosin increased with the increase in its concentration, while its antioxidant activity is still at an over low level compared to that of Vitamin C; for instance, the DPPH scavenging activities of 7.5 μg/mL maculosin and 7.5 μg/mL Vitamin C were 38.22% and 74.44%, respectively (Figure 7B).

## 4. Discussion

Marine bacteria have been regarded as valuable sources for novel bioactivity metabolites with better biocatalytic properties, e.g., high salinity, high pressure, hyperthermostability, barophilicity, alkali resistance, and low temperature, which can serve various biotechnological applications, such as pharmaceutical, food, and cosmetics [14,18,22,23,24,26,27,28]. Agar in the macroalgae accounted for ~60% of dry weight in its cell wall [29], and the bacteria with the capacity of metabolizing agar as their only carbon and energy source are recognized as agarolytic bacteria. Additionally, unique enzymes and metabolites with unpreceded applications in industry and medicine have been derived from agarolytic bacteria [3,30,31].

In the present work, an agarolytic bacterium strain QZ9-9 was successfully isolated from tropical *G. hainanensis* in Hainan Island, China, which was classified into the species of *Alteromonas macleodii* (Gene Bank No.: CP098772.1). *A. macleodii* QZ9-9 grew well in either high nutrient medium or only carbon source medium, whereas the agarase secreted by the strain QZ9-9 must be inducible by agar. The production process of the substrate (agar)-induced agarase of *A. macleodii* QZ9-9 is similar to that of many other induced enzymes, such as dextranase induced by dextran [9,18], β-galactosidase induced by lactose or galactose [32], α-amylase induced by amylum [33], and agarase induced by agar [14,34,35]. 

*A. macleodii* QZ9-9 displayed a particularly strong ability to degrade agar. With the increase in culture time, the diameter of the degradation zone generated by this strain also increased, and the maximum diameter of the degradation zone reached 41.46 mm when the culture time was 108 h, and the first-order degradation rate constant was about 0.02 h^−^^1^. As expected, the degradation capacity of the crude agarase of *A. macleodii* QZ9-9 also increased with the increase in enzyme activity. When the enzyme activity reached 12.16 U/mL, the maximum diameter of the degradation zone was up to about 22.89 mm, and the first-order degradation rate constant was about 0.77 U^−1^. Only a few species belonging to the *Alteromonas*-like Gammaproteobacteria are reported to be agarolytic or weakly agarolytic, such as *Pseudoalteromonas* sp. [36,37], *Shewanella* sp. [38,39], and *Glaciecola* sp. [40], whereas our results indicated that *A. macleodii* QZ9-9 is a good candidate for agarolytic bacteria with a strong agar-degrading capacity. 

In particular, the fermentation broth of *A. macleodii* QZ9-9 was found to possess high antioxidant activity, and the peak of DPPH scavenging activity of its fermentation products after culture for 50 h was up to 50.79% in the reaction for 1 h. Furthermore, the low molecule metabolites (≤3 kDa) of *A. macleodii* QZ9-9 after incubation for 50 h displayed the highest DPPH scavenging activity of 85.85%, which contained a total of 766 metabolites. Among them, most metabolites (217 metabolites) were identified as amino acids/peptides/analogs. More importantly, the peptide-like metabolites, such as prolyl–histidine, isoleucyl–histidine, isoleucyl–proline, and arginyl–proline, were found in the top 20 metabolites with high abundance.

Reportedly, antioxidants are compounds that stop the oxidation process by inhibiting free radical reactions, and the antioxidants protect against the damaging effects of metabolism in biological systems, such as neutralizing excess free radicals in the bio-body. There are many kinds of antioxidant substances in the ocean, mainly including polysaccharides, terpenes, polyphenolic compounds, enzymatic antioxidants, amino acids, and peptides. Of the antioxidants, peptides consisting of 2–20 amino acid residues were frequently reported due to their antioxidant activities, such as glutathione, carnosine, cyclo (His-Pro), and human tripeptide GHK (glycyl-*l*-histidyl-Llysine) [41], and the antioxidant activity of antioxidant peptides are mainly related to the arrangement of amino acid and the structure of the peptide. Additionally, the study on the antibacterial, anticancer, anti-inflammatory, and other biological activities of antioxidant peptides is also of high research interest [42]. The marine sources of antioxidant peptides are varied, such as algae, sponges, bryozoans, lichens, microalgae, bacteria, etc., which are all natural sources of antioxidants [43]; for instance, Tripathi et al. [44] found that short peptides in *Kocuria Marina* CDMP 10 extract were effective in reducing DPPH free radicals during antioxidant molecular production screening of marine microbial resources in the Bay of Bengal, India. Sonani et al. [45] isolated three water-soluble phycobilin proteins: phycoerythrin, phycocyanin, and allophycocyanin from the cyanobacteria *Lyngbya* sp. A09DM, which showed strong antioxidant and free radical scavenging activities and could be used to prolong the life span of *Caenorhabditis elegans*. Abuine et al. [46] found that short peptides purified from fish skin hydrolysates, in addition to exhibiting antioxidant activity, also displayed hypoglycemic, antihypertensive, antibacterial, and anti-aging activities. In this work, we reason that the low molecule metabolites (≤3 kDa) of *A. macleodii* QZ9-9 containing high peptide contents may be a strong candidate to screen and prepare antioxidant peptides due to its relatively high antioxidant activity. 

Additionally, we focused on a maculosin (C_14_H_16_N_2_O_3_) metabolite in the low molecule metabolites (≤3 kDa) of *A. macleodii* QZ9-9 due to its relatively high abundance. Maculosin was first reported in 1988 as a host-specific phytotoxin for spotted knapweed produced by *Alternaria alternata* [47]. After that, it was successively isolated from *Schizophyllum commune* [48], *Pseudomonas* sp. (ABS-36) [49], *Streptomyces* sp. ZZ446 [50], *Bacillus pumilus* AMK1 [51], and *Streptomyces* sp. KTM18 [52]. In terms of the bioactivity of maculosin, there were only a few reports on the properties of the bioactive compound; for instance, the maculosin isolated from *Streptomyces* sp. ZZ446 showed good antibacterial activity against methicillin-resistant *Staphylococcus aureus*, *Escherichia coli*, and *Candida albicans* [50]; Karanam et al. [51] reported that the maculosin played an anticancer role by inducing apoptosis. In terms of antioxidant activity, the maculosin isolated from *Streptomyces* sp. KTM18 displayed antioxidant activity [52]. Given this, the antioxidant activity of maculosin was furtherly confirmed by the method of DPPH scavenging activity in this work. We found that although the DPPH scavenging activity of the maculosin was weaker than that of Vitamin C, the obvious scavenging ability for DPPH was visible after treatment of maculosin, and the purple of DPPH solution gradually faded to lavender with the increase in maculosin concentration. When the concentration of maculosin increased to 750 μg/mL, the DPPH scavenging activity was up to about 56.89%, indicating that maculosin has a certain antioxidant effect. In addition, maculosin was reported to be a non-toxic substance to a certain extent [51,52]. Therefore, we reason that the maculosin-enriched low molecule metabolites (≤3 kDa) of *A. macleodii* QZ9-9 with relatively high antioxidant activity must be the strong candidates for preparing antioxidant maculosin.

## 5. Conclusions

In summary, the agarolytic bacterium-*A. macleodii* QZ9-9 isolated from tropical *G. hainanensis* in Hainan Island was a strong candidate for utilization of macroalgae resources by degrading the agar that is the main component of macroalgae. In particular, the low molecule metabolites (≤3 kDa) of *A. macleodii* QZ9-9 with relatively high antioxidant activity are strong candidates for preparing non-toxic antioxidants such as antioxidant peptides and maculosin. Therefore, we reason that the agarolytic bacterium-*A. macleodii* QZ9-9 and its metabolites/degradants are possibilities for high-added value utilization of macroalgae in the fields of cosmetics, food preservation, and the pharmaceutical industry.

## Figures and Tables

**Table 1:** The metabolites of the low molecular weight fermentation products of *A. macleodii* QZ9-9 with the relative abundances of the top 20.

No.	Metabolite	m/z	Retention Time (min)	Formula	KEGG ID	Library ID	Area (Counts)	Relative Abundance (%)
1	Meso–Pristane	332.33	10.87	C_19_H_40_	-	HMDB0034497	5.39 × 10^9^	54.96%
2	Prolyl–Histidine	252.00	2.91	C_11_H_16_N_4_O_3_	-	HMDB0029019	2.63 × 10^8^	2.68%
3	Hexadecasphinganine	273.27	10.20	C_16_H_35_NO_2_	C13915	LMSP01040001	1.54 × 10^8^	1.58%
4	Glycerol	115.04	1.01	C_3_H_8_O_3_	C00116	HMDB0000131; 56-81-5	1.17 × 10^8^	1.20%
5	6-Hydroxyoctadecanoic acid	318.30	10.21	C_18_H_36_O_3_	-	HMDB0112193	1.10 × 10^8^	1.12%
6	Maculosin	261.12	5.90	C_14_H_16_N_2_O_3_	C10605	-	9.28 × 10^7^	0.95%
7	Benzalkonium	304.30	10.49	C_21_H_37_N	-	-	7.91 × 10^7^	0.81%
8	MG(0:0/16:0/0:0)	353.27	12.680	C_19_H_38_O_4_	-	HMDB0011533; LMGL01010025	5.28 × 10^7^	0.54%
9	(2xi,6xi)-7-Methyl-3-methylene-1,2,6,7-octanetetrol	227.13	9.63	C_10_H_20_O_4_	-	HMDB0033217; LMFA05000554	4.71 × 10^7^	0.48%
10	10,20-Dihydroxyeicosanoic acid	362.33	10.22	C_20_H_40_O_4_	-	HMDB0031923	4.04 × 10^7^	0.41%
11	(+/−)-Flurbiprofen	245.10	4.43	C_15_H_13_FO_2_	-	-	3.69 × 10^7^	0.38%
12	2-Isopropyl-3,5-dimethoxy-6-methylpyrazine	197.13	6.26	C_10_H_16_N_2_O_2_	-	HMDB0029741	3.54 × 10^7^	0.36%
13	Acrylamide	181.04	16.30	C_3_H_5_NO	C01659	HMDB0004296	3.41 × 10^7^	0.35%
14	Isoleucyl–Histidine	251.15	3.94	C_12_H_20_N_4_O_3_	-	HMDB0028909	2.90 × 10^7^	0.30%
15	4-(3,7-dimethylocta-2,6-dien-1-yl)benzene-1,2,3,5-tetrol	301.14	11.45	C_16_H_22_O_4_	-	-	2.88 × 10^7^	0.29%
16	Isoleucyl–proline	211.14	7.34	C_11_H_20_N_2_O_3_	-	HMDB0011174	2.80 × 10^7^	0.29%
17	Arginyl–Proline	254.16	3.73	C_11_H_21_N_5_O_3_	-	HMDB0028717	2.62 × 10^7^	0.27%
18	1-(4-hydroxy-3-methoxyphenyl)-7-(3-hydroxyphenyl)heptane-3,5-dione	365.14	11.38	C_20_H_22_O_5_	-	-	2.45 × 10^7^	0.25%
19	2-Deoxyglucose	187.06	1.04	C_6_H_12_O_5_	-	HMDB0062477	2.30 × 10^7^	0.23%
20	Taurocholic acid	538.28	11.42	C_26_H_45_NO_7_S	C05122	81-24-3; LMST05040001; HMDB0000036	2.28 × 10^7^	0.23%

## Data Availability

The data presented in this study are available in the article and Supplementary Materials.

## Supplementary Materials

The following supporting information can be downloaded at: https://www.mdpi.com/article/10.3390/antiox11101977/s1.
